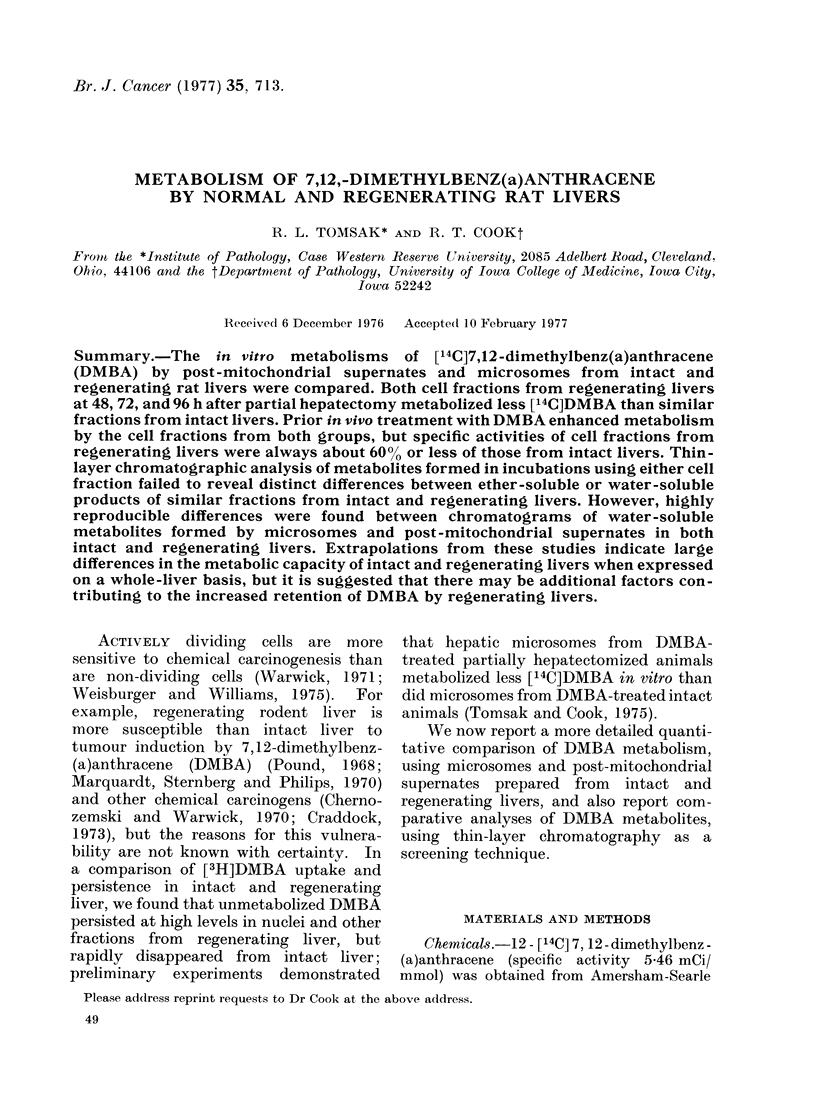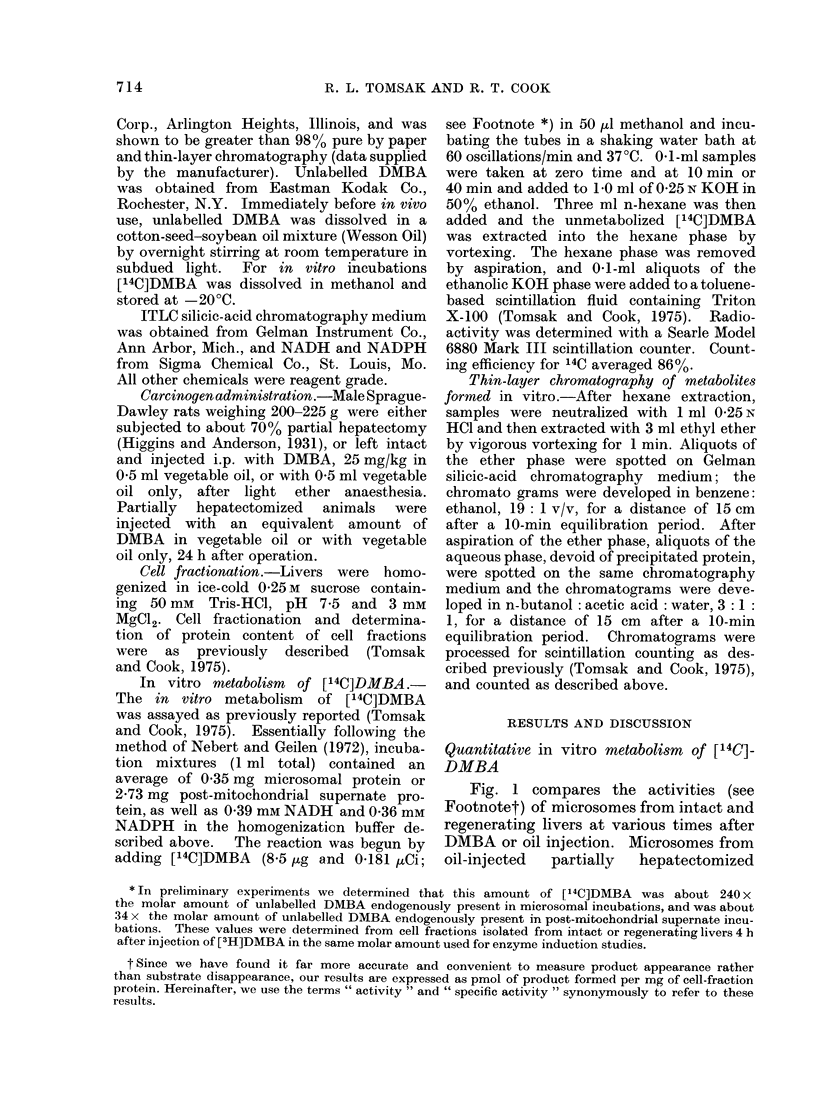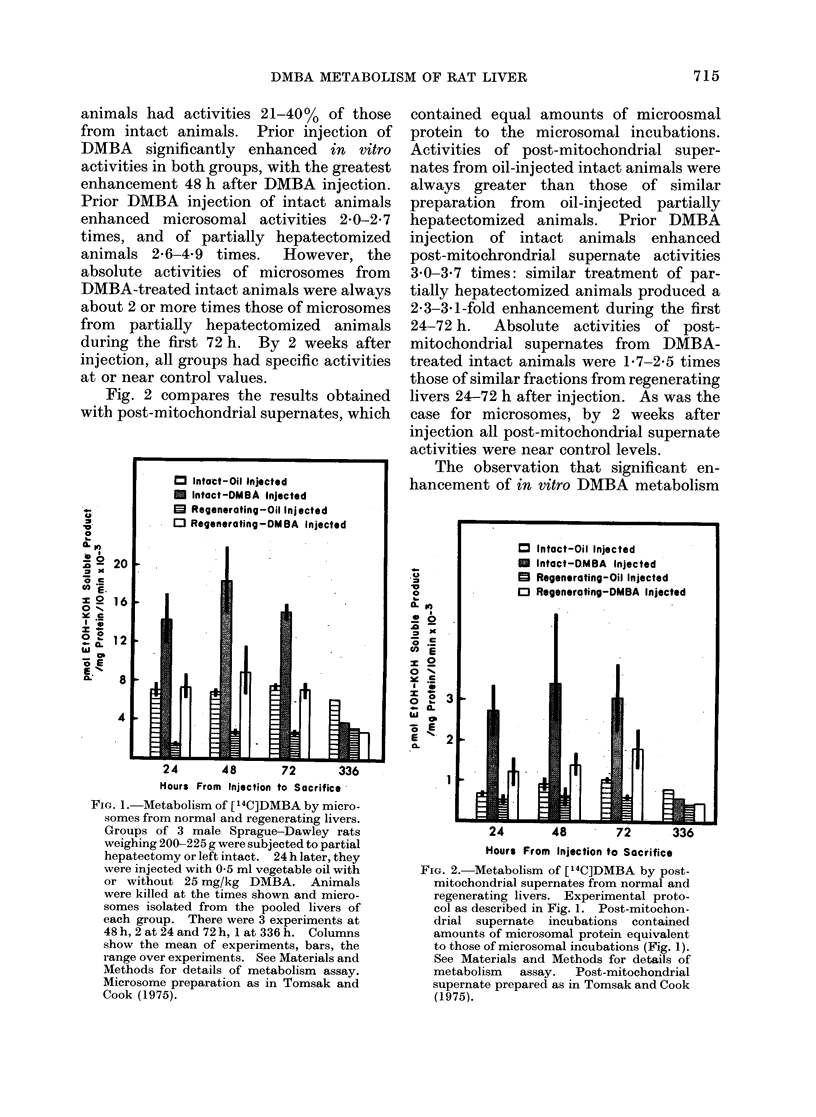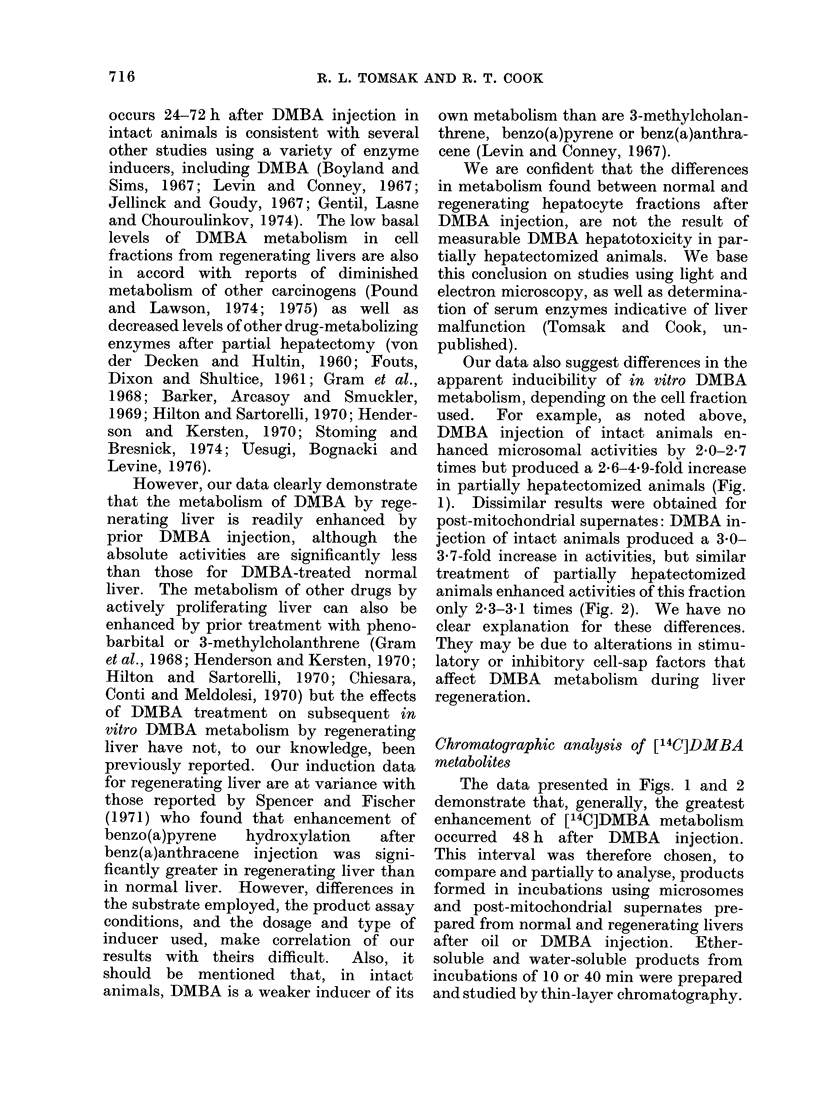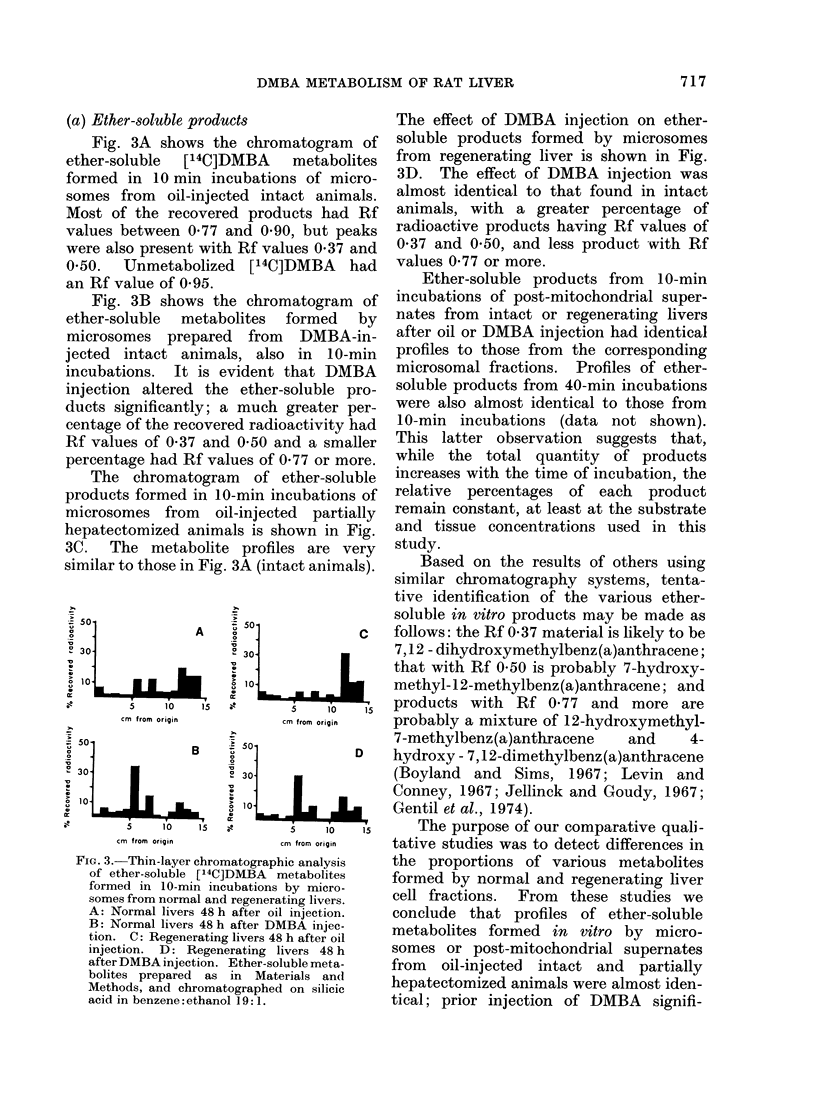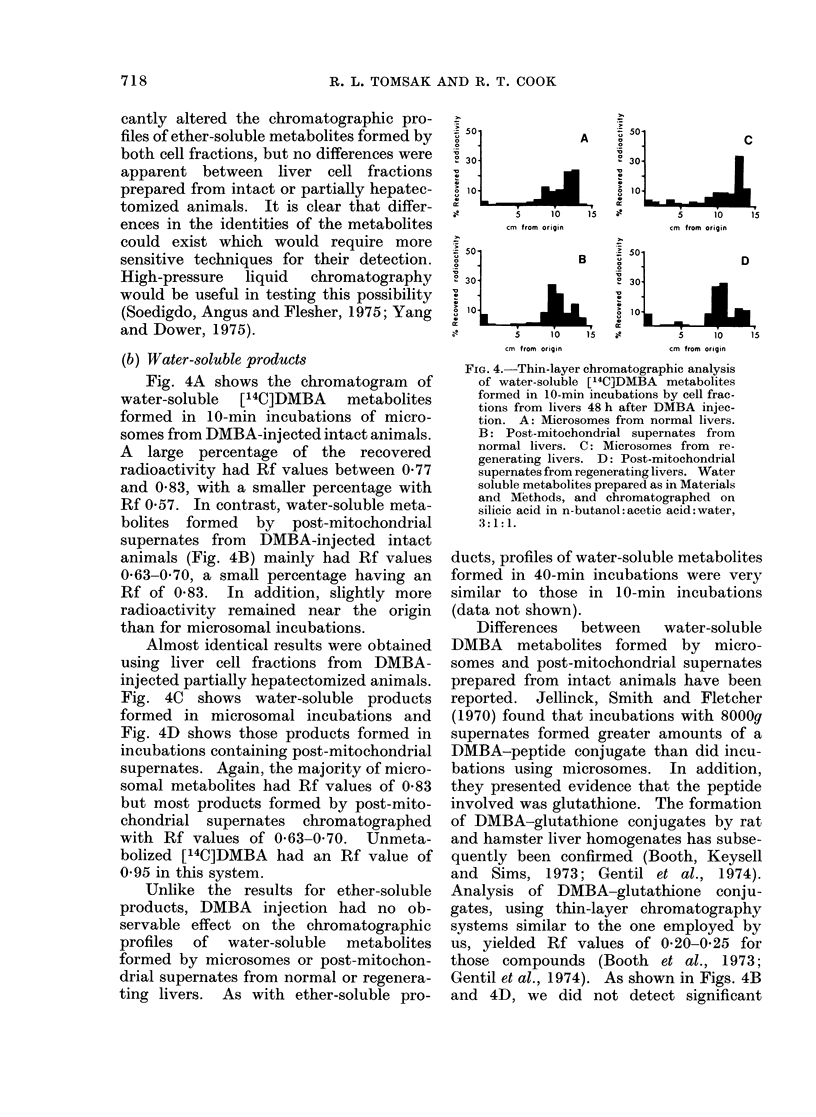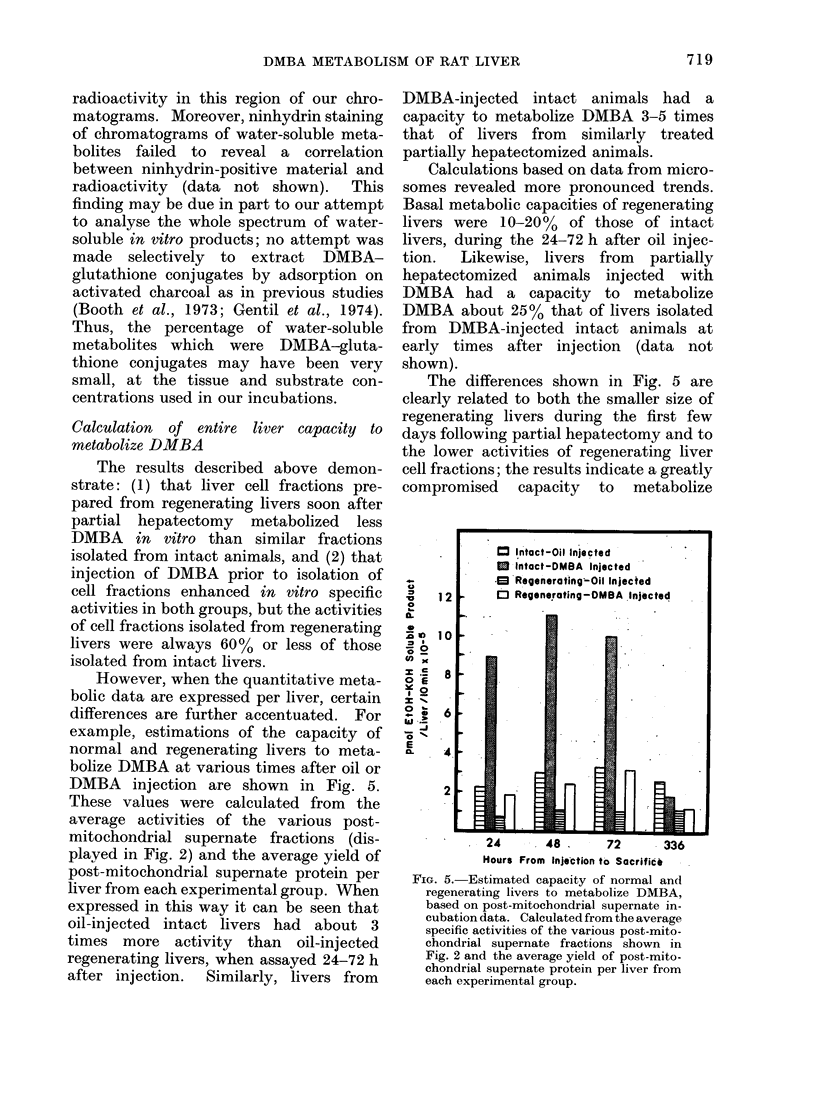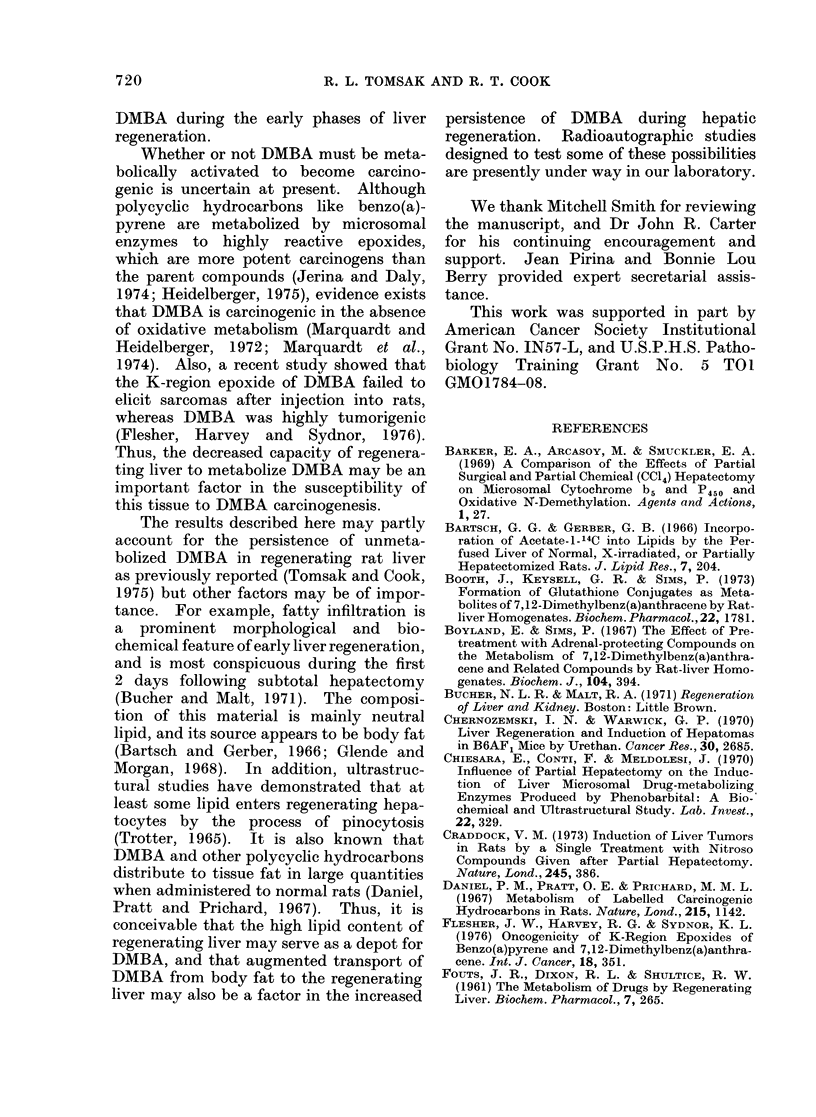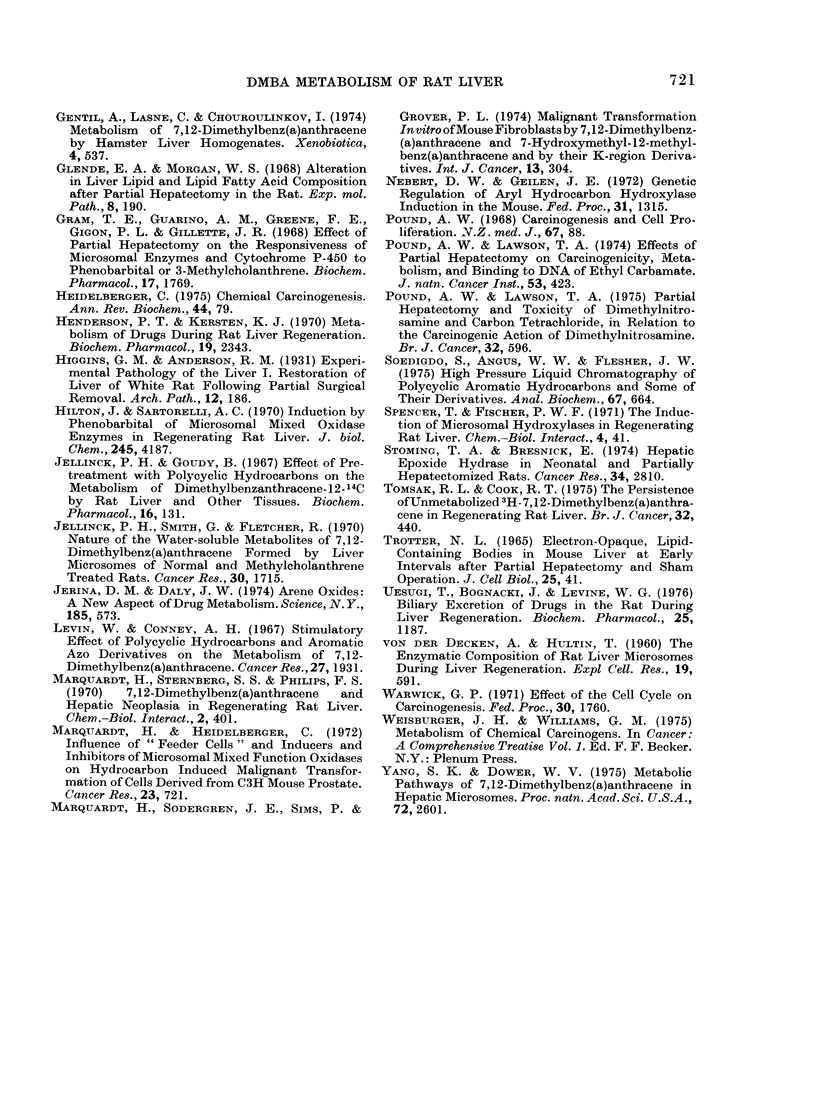# Metabolism of 7,12,-dimethylbenz(a)anthracene by normal and regenerating rat livers.

**DOI:** 10.1038/bjc.1977.112

**Published:** 1977-06

**Authors:** R. L. Tomsak, R. T. Cook

## Abstract

The in vitro metabolisms of [14C]7,12-dimethylbenz(a)anthracene (DMBA) by post-mitochondrial supernates and microsomes from intact and regenerating rat livers were compared. Both cell fractions from regenerating livers at 48, 72, and 96 h after partial hepatectomy metabolized less [14C]DMBA than similar fractions from intact livers. Prior in vivo treatment with DMBA enhanced metabolism by the cell fractions from both groups, but specific activities of cell fractions from regenerating livers were always about 60% or less of those from intact livers. Thin-layer chromatographic analysis of metabolites formed in incubations using either cell fraction failed to reveal distinct differences between ether-soluble or water-soluble products of similar fractions from intact and regenerating livers. However, highly reproducible differences were found between chromatograms of water-soluble metabolites formed by microsomes and post-mitochondrial supernates in both intact and regenerating livers. Extrapolations from these studies indicate large differences in the metabolic capacity of intact and regenerating livers when expressed on a whole-liver basis, but it is suggested that there may be additional factors contributing to the increased retention of DMBA by regenerating livers.


Br. J. Cancer (1977) 35, 713.

### METABOLISM OF 7,12,-DIMETHYLBENZ(a)ANTHRACENE BY NORMAL AND REGENERATING RAT LIVERS

R. L. TOMSAK* AND R. T. COOK†

*From the *Institute of Pathology, Case Western Reserve University,* 2085 *Adelbert Road, Cleveland, Ohio,* 44106 *and the †Department of Pathology, University of Iowa College of Medicine, Iowa City, Iowa* 52242

Received 6 December 1976 Accepted 10 February 1977

**Summary.—The *in vitro* metabolisms of [¹⁴C]7,12-dimethylbenz(a)anthracene (DMBA) by post-mitochondrial supernates and microsomes from intact and regenerating rat livers were compared. Both cell fractions from regenerating livers at 48, 72, and 96 h after partial hepatectomy metabolized less [¹⁴C]DMBA than similar fractions from intact livers. Prior *in vivo* treatment with DMBA enhanced metabolism by the cell fractions from both groups, but specific activities of cell fractions from regenerating livers were always about 60% or less of those from intact livers. Thin-layer chromatographic analysis of metabolites formed in incubations using either cell fraction failed to reveal distinct differences between ether-soluble or water-soluble products of similar fractions from intact and regenerating livers. However, highly reproducible differences were found between chromatograms of water-soluble metabolites formed by microsomes and post-mitochondrial supernates in both intact and regenerating livers. Extrapolations from these studies indicate large differences in the metabolic capacity of intact and regenerating livers when expressed on a whole-liver basis, but it is suggested that there may be additional factors contributing to the increased retention of DMBA by regenerating livers.**

ACTIVELY dividing cells are more sensitive to chemical carcinogenesis than are non-dividing cells (Warwick, 1971; Weisburger and Williams, 1975). For example, regenerating rodent liver is more susceptible than intact liver to tumour induction by 7,12-dimethylbenz-(a)anthracene (DMBA) (Pound, 1968; Marquardt, Sternberg and Philips, 1970) and other chemical carcinogens (Chernozemski and Warwick, 1970; Craddock, 1973), but the reasons for this vulnerability are not known with certainty. In a comparison of [$^3$H]DMBA uptake and persistence in intact and regenerating liver, we found that unmetabolized DMBA persisted at high levels in nuclei and other fractions from regenerating liver, but rapidly disappeared from intact liver; preliminary experiments demonstrated that hepatic microsomes from DMBA-treated partially hepatectomized animals metabolized less [$^{14}$C]DMBA *in vitro* than did microsomes from DMBA-treated intact animals (Tomsak and Cook, 1975).

We now report a more detailed quantitative comparison of DMBA metabolism, using microsomes and post-mitochondrial supernates prepared from intact and regenerating livers, and also report comparative analyses of DMBA metabolites, using thin-layer chromatography as a screening technique.

### MATERIALS AND METHODS

*Chemicals.*—12-[$^{14}$C]7,12-dimethylbenz-(a)anthracene (specific activity 5·46 mCi/mmol) was obtained from Amersham-Searle

Please address reprint requests to Dr Cook at the above address.

Corp., Arlington Heights, Illinois, and was shown to be greater than 98% pure by paper and thin-layer chromatography (data supplied by the manufacturer). Unlabelled DMBA was obtained from Eastman Kodak Co., Rochester, N.Y. Immediately before *in vivo* use, unlabelled DMBA was dissolved in a cotton-seed–soybean oil mixture (Wesson Oil) by overnight stirring at room temperature in subdued light. For *in vitro* incubations [$^{14}$C]DMBA was dissolved in methanol and stored at −20°C.

ITLC silicic-acid chromatography medium was obtained from Gelman Instrument Co., Ann Arbor, Mich., and NADH and NADPH from Sigma Chemical Co., St. Louis, Mo. All other chemicals were reagent grade.

*Carcinogen administration.*—Male Sprague-Dawley rats weighing 200–225 g were either subjected to about 70% partial hepatectomy (Higgins and Anderson, 1931), or left intact and injected i.p. with DMBA, 25 mg/kg in 0·5 ml vegetable oil, or with 0·5 ml vegetable oil only, after light ether anaesthesia. Partially hepatectomized animals were injected with an equivalent amount of DMBA in vegetable oil or with vegetable oil only, 24 h after operation.

*Cell fractionation.*—Livers were homogenized in ice-cold 0·25 M sucrose containing 50 mM Tris-HCl, pH 7·5 and 3 mM $MgCl_2$. Cell fractionation and determination of protein content of cell fractions were as previously described (Tomsak and Cook, 1975).

In vitro *metabolism of* [$^{14}$C]*DMBA*.—The *in vitro* metabolism of [$^{14}$C]DMBA was assayed as previously reported (Tomsak and Cook, 1975). Essentially following the method of Nebert and Geilen (1972), incubation mixtures (1 ml total) contained an average of 0·35 mg microsomal protein or 2·73 mg post-mitochondrial supernate protein, as well as 0·39 mM NADH and 0·36 mM NADPH in the homogenization buffer described above. The reaction was begun by adding [$^{14}$C]DMBA (8·5 μg and 0·181 μCi; see Footnote *) in 50 μl methanol and incubating the tubes in a shaking water bath at 60 oscillations/min and 37°C. 0·1-ml samples were taken at zero time and at 10 min or 40 min and added to 1·0 ml of 0·25 N KOH in 50% ethanol. Three ml n-hexane was then added and the unmetabolized [$^{14}$C]DMBA was extracted into the hexane phase by vortexing. The hexane phase was removed by aspiration, and 0·1-ml aliquots of the ethanolic KOH phase were added to a toluene-based scintillation fluid containing Triton X-100 (Tomsak and Cook, 1975). Radioactivity was determined with a Searle Model 6880 Mark III scintillation counter. Counting efficiency for $^{14}$C averaged 86%.

*Thin-layer chromatography of metabolites formed* in vitro.—After hexane extraction, samples were neutralized with 1 ml 0·25 N HCl and then extracted with 3 ml ethyl ether by vigorous vortexing for 1 min. Aliquots of the ether phase were spotted on Gelman silicic-acid chromatography medium; the chromato grams were developed in benzene: ethanol, 19 : 1 v/v, for a distance of 15 cm after a 10-min equilibration period. After aspiration of the ether phase, aliquots of the aqueous phase, devoid of precipitated protein, were spotted on the same chromatography medium and the chromatograms were developed in n-butanol : acetic acid : water, 3 : 1 : 1, for a distance of 15 cm after a 10-min equilibration period. Chromatograms were processed for scintillation counting as described previously (Tomsak and Cook, 1975), and counted as described above.

### RESULTS AND DISCUSSION

### *Quantitative* in vitro *metabolism of* [$^{14}$*C*]-*DMBA*

Fig. 1 compares the activities (see Footnote†) of microsomes from intact and regenerating livers at various times after DMBA or oil injection. Microsomes from oil-injected partially hepatectomized

* In preliminary experiments we determined that this amount of [$^{14}$C]DMBA was about 240× the molar amount of unlabelled DMBA endogenously present in microsomal incubations, and was about 34× the molar amount of unlabelled DMBA endogenously present in post-mitochondrial supernate incubations. These values were determined from cell fractions isolated from intact or regenerating livers 4 h after injection of [$^{3}$H]DMBA in the same molar amount used for enzyme induction studies.

† Since we have found it far more accurate and convenient to measure product appearance rather than substrate disappearance, our results are expressed as pmol of product formed per mg of cell-fraction protein. Hereinafter, we use the terms " activity " and " specific activity " synonymously to refer to these results.

animals had activities 21–40% of those from intact animals. Prior injection of DMBA significantly enhanced *in vitro* activities in both groups, with the greatest enhancement 48 h after DMBA injection. Prior DMBA injection of intact animals enhanced microsomal activities 2·0–2·7 times, and of partially hepatectomized animals 2·6–4·9 times. However, the absolute activities of microsomes from DMBA-treated intact animals were always about 2 or more times those of microsomes from partially hepatectomized animals during the first 72 h. By 2 weeks after injection, all groups had specific activities at or near control values.

Fig. 2 compares the results obtained with post-mitochondrial supernates, which contained equal amounts of microosmal protein to the microsomal incubations. Activities of post-mitochondrial supernates from oil-injected intact animals were always greater than those of similar preparation from oil-injected partially hepatectomized animals. Prior DMBA injection of intact animals enhanced post-mitochrondrial supernate activities 3·0–3·7 times: similar treatment of partially hepatectomized animals produced a 2·3–3·1-fold enhancement during the first 24–72 h. Absolute activities of post-mitochondrial supernates from DMBA-treated intact animals were 1·7–2·5 times those of similar fractions from regenerating livers 24–72 h after injection. As was the case for microsomes, by 2 weeks after injection all post-mitochondrial supernate activities were near control levels.

The observation that significant enhancement of *in vitro* DMBA metabolism

FIG. 1.—Metabolism of [$^{14}$C]DMBA by microsomes from normal and regenerating livers. Groups of 3 male Sprague–Dawley rats weighing 200–225 g were subjected to partial hepatectomy or left intact. 24 h later, they were injected with 0·5 ml vegetable oil with or without 25 mg/kg DMBA. Animals were killed at the times shown and microsomes isolated from the pooled livers of each group. There were 3 experiments at 48 h, 2 at 24 and 72 h, 1 at 336 h. Columns show the mean of experiments, bars, the range over experiments. See Materials and Methods for details of metabolism assay. Microsome preparation as in Tomsak and Cook (1975).

FIG. 2.—Metabolism of [$^{14}$C]DMBA by post-mitochondrial supernates from normal and regenerating livers. Experimental protocol as described in Fig. 1. Post-mitochondrial supernate incubations contained amounts of microsomal protein equivalent to those of microsomal incubations (Fig. 1). See Materials and Methods for details of metabolism assay. Post-mitochondrial supernate prepared as in Tomsak and Cook (1975).

occurs 24–72 h after DMBA injection in intact animals is consistent with several other studies using a variety of enzyme inducers, including DMBA (Boyland and Sims, 1967; Levin and Conney, 1967; Jellinck and Goudy, 1967; Gentil, Lasne and Chouroulinkov, 1974). The low basal levels of DMBA metabolism in cell fractions from regenerating livers are also in accord with reports of diminished metabolism of other carcinogens (Pound and Lawson, 1974; 1975) as well as decreased levels of other drug-metabolizing enzymes after partial hepatectomy (von der Decken and Hultin, 1960; Fouts, Dixon and Shultice, 1961; Gram *et al.*, 1968; Barker, Arcasoy and Smuckler, 1969; Hilton and Sartorelli, 1970; Henderson and Kersten, 1970; Stoming and Bresnick, 1974; Uesugi, Bognacki and Levine, 1976).

However, our data clearly demonstrate that the metabolism of DMBA by regenerating liver is readily enhanced by prior DMBA injection, although the absolute activities are significantly less than those for DMBA-treated normal liver. The metabolism of other drugs by actively proliferating liver can also be enhanced by prior treatment with phenobarbital or 3-methylcholanthrene (Gram *et al.*, 1968; Henderson and Kersten, 1970; Hilton and Sartorelli, 1970; Chiesara, Conti and Meldolesi, 1970) but the effects of DMBA treatment on subsequent *in vitro* DMBA metabolism by regenerating liver have not, to our knowledge, been previously reported. Our induction data for regenerating liver are at variance with those reported by Spencer and Fischer (1971) who found that enhancement of benzo(a)pyrene hydroxylation after benz(a)anthracene injection was significantly greater in regenerating liver than in normal liver. However, differences in the substrate employed, the product assay conditions, and the dosage and type of inducer used, make correlation of our results with theirs difficult. Also, it should be mentioned that, in intact animals, DMBA is a weaker inducer of its own metabolism than are 3-methylcholanthrene, benzo(a)pyrene or benz(a)anthracene (Levin and Conney, 1967).

We are confident that the differences in metabolism found between normal and regenerating hepatocyte fractions after DMBA injection, are not the result of measurable DMBA hepatotoxicity in partially hepatectomized animals. We base this conclusion on studies using light and electron microscopy, as well as determination of serum enzymes indicative of liver malfunction (Tomsak and Cook, unpublished).

Our data also suggest differences in the apparent inducibility of *in vitro* DMBA metabolism, depending on the cell fraction used. For example, as noted above, DMBA injection of intact animals enhanced microsomal activities by 2·0–2·7 times but produced a 2·6–4·9-fold increase in partially hepatectomized animals (Fig. 1). Dissimilar results were obtained for post-mitochondrial supernates: DMBA injection of intact animals produced a 3·0–3·7-fold increase in activities, but similar treatment of partially hepatectomized animals enhanced activities of this fraction only 2·3–3·1 times (Fig. 2). We have no clear explanation for these differences. They may be due to alterations in stimulatory or inhibitory cell-sap factors that affect DMBA metabolism during liver regeneration.

### *Chromatographic analysis of [$^{14}$C]DMBA metabolites*

The data presented in Figs. 1 and 2 demonstrate that, generally, the greatest enhancement of [$^{14}$C]DMBA metabolism occurred 48 h after DMBA injection. This interval was therefore chosen, to compare and partially to analyse, products formed in incubations using microsomes and post-mitochondrial supernates prepared from normal and regenerating livers after oil or DMBA injection. Ether-soluble and water-soluble products from incubations of 10 or 40 min were prepared and studied by thin-layer chromatography.

(*a*) *Ether-soluble products*

Fig. 3A shows the chromatogram of ether-soluble [$^{14}$C]DMBA metabolites formed in 10 min incubations of microsomes from oil-injected intact animals. Most of the recovered products had Rf values between 0·77 and 0·90, but peaks were also present with Rf values 0·37 and 0·50. Unmetabolized [$^{14}$C]DMBA had an Rf value of 0·95.

Fig. 3B shows the chromatogram of ether-soluble metabolites formed by microsomes prepared from DMBA-injected intact animals, also in 10-min incubations. It is evident that DMBA injection altered the ether-soluble products significantly; a much greater percentage of the recovered radioactivity had Rf values of 0·37 and 0·50 and a smaller percentage had Rf values of 0·77 or more.

The chromatogram of ether-soluble products formed in 10-min incubations of microsomes from oil-injected partially hepatectomized animals is shown in Fig. 3C. The metabolite profiles are very similar to those in Fig. 3A (intact animals).

FIG. 3.—Thin-layer chromatographic analysis of ether-soluble [$^{14}$C]DMBA metabolites formed in 10-min incubations by microsomes from normal and regenerating livers. A: Normal livers 48 h after oil injection. B: Normal livers 48 h after DMBA injection. C: Regenerating livers 48 h after oil injection. D: Regenerating livers 48 h after DMBA injection. Ether-soluble metabolites prepared as in Materials and Methods, and chromatographed on silicic acid in benzene:ethanol 19:1.

The effect of DMBA injection on ether-soluble products formed by microsomes from regenerating liver is shown in Fig. 3D. The effect of DMBA injection was almost identical to that found in intact animals, with a greater percentage of radioactive products having Rf values of 0·37 and 0·50, and less product with Rf values 0·77 or more.

Ether-soluble products from 10-min incubations of post-mitochondrial supernates from intact or regenerating livers after oil or DMBA injection had identical profiles to those from the corresponding microsomal fractions. Profiles of ether-soluble products from 40-min incubations were also almost identical to those from 10-min incubations (data not shown). This latter observation suggests that, while the total quantity of products increases with the time of incubation, the relative percentages of each product remain constant, at least at the substrate and tissue concentrations used in this study.

Based on the results of others using similar chromatography systems, tentative identification of the various ether-soluble *in vitro* products may be made as follows: the Rf 0·37 material is likely to be 7,12-dihydroxymethylbenz(a)anthracene; that with Rf 0·50 is probably 7-hydroxymethyl-12-methylbenz(a)anthracene; and products with Rf 0·77 and more are probably a mixture of 12-hydroxymethyl-7-methylbenz(a)anthracene and 4-hydroxy-7,12-dimethylbenz(a)anthracene (Boyland and Sims, 1967; Levin and Conney, 1967; Jellinck and Goudy, 1967; Gentil *et al.*, 1974).

The purpose of our comparative qualitative studies was to detect differences in the proportions of various metabolites formed by normal and regenerating liver cell fractions. From these studies we conclude that profiles of ether-soluble metabolites formed *in vitro* by microsomes or post-mitochondrial supernates from oil-injected intact and partially hepatectomized animals were almost identical; prior injection of DMBA significantly altered the chromatographic profiles of ether-soluble metabolites formed by both cell fractions, but no differences were apparent between liver cell fractions prepared from intact or partially hepatectomized animals. It is clear that differences in the identities of the metabolites could exist which would require more sensitive techniques for their detection. High-pressure liquid chromatography would be useful in testing this possibility (Soedigdo, Angus and Flesher, 1975; Yang and Dower, 1975).

(*b*) *Water-soluble products*

Fig. 4A shows the chromatogram of water-soluble [$^{14}$C]DMBA metabolites formed in 10-min incubations of microsomes from DMBA-injected intact animals. A large percentage of the recovered radioactivity had Rf values between 0·77 and 0·83, with a smaller percentage with Rf 0·57. In contrast, water-soluble metabolites formed by post-mitochondrial supernates from DMBA-injected intact animals (Fig. 4B) mainly had Rf values 0·63–0·70, a small percentage having an Rf of 0·83. In addition, slightly more radioactivity remained near the origin than for microsomal incubations.

Almost identical results were obtained using liver cell fractions from DMBA-injected partially hepatectomized animals. Fig. 4C shows water-soluble products formed in microsomal incubations and Fig. 4D shows those products formed in incubations containing post-mitochondrial supernates. Again, the majority of microsomal metabolites had Rf values of 0·83 but most products formed by post-mitochondrial supernates chromatographed with Rf values of 0·63–0·70. Unmetabolized [$^{14}$C]DMBA had an Rf value of 0·95 in this system.

Unlike the results for ether-soluble products, DMBA injection had no observable effect on the chromatographic profiles of water-soluble metabolites formed by microsomes or post-mitochondrial supernates from normal or regenerating livers. As with ether-soluble products, profiles of water-soluble metabolites formed in 40-min incubations were very similar to those in 10-min incubations (data not shown).

FIG. 4.—Thin-layer chromatographic analysis of water-soluble [$^{14}$C]DMBA metabolites formed in 10-min incubations by cell fractions from livers 48 h after DMBA injection. A: Microsomes from normal livers. B: Post-mitochondrial supernates from normal livers. C: Microsomes from regenerating livers. D: Post-mitochondrial supernates from regenerating livers. Water soluble metabolites prepared as in Materials and Methods, and chromatographed on silicic acid in n-butanol:acetic acid:water, 3:1:1.

Differences between water-soluble DMBA metabolites formed by microsomes and post-mitochondrial supernates prepared from intact animals have been reported. Jellinck, Smith and Fletcher (1970) found that incubations with 8000*g* supernates formed greater amounts of a DMBA–peptide conjugate than did incubations using microsomes. In addition, they presented evidence that the peptide involved was glutathione. The formation of DMBA–glutathione conjugates by rat and hamster liver homogenates has subsequently been confirmed (Booth, Keysell and Sims, 1973; Gentil *et al.*, 1974). Analysis of DMBA–glutathione conjugates, using thin-layer chromatography systems similar to the one employed by us, yielded Rf values of 0·20–0·25 for those compounds (Booth *et al.*, 1973; Gentil *et al.*, 1974). As shown in Figs. 4B and 4D, we did not detect significant radioactivity in this region of our chromatograms. Moreover, ninhydrin staining of chromatograms of water-soluble metabolites failed to reveal a correlation between ninhydrin-positive material and radioactivity (data not shown). This finding may be due in part to our attempt to analyse the whole spectrum of water-soluble *in vitro* products; no attempt was made selectively to extract DMBA–glutathione conjugates by adsorption on activated charcoal as in previous studies (Booth *et al.*, 1973; Gentil *et al.*, 1974). Thus, the percentage of water-soluble metabolites which were DMBA–glutathione conjugates may have been very small, at the tissue and substrate concentrations used in our incubations.

*Calculation of entire liver capacity to metabolize DMBA*

The results described above demonstrate: (1) that liver cell fractions prepared from regenerating livers soon after partial hepatectomy metabolized less DMBA *in vitro* than similar fractions isolated from intact animals, and (2) that injection of DMBA prior to isolation of cell fractions enhanced *in vitro* specific activities in both groups, but the activities of cell fractions isolated from regenerating livers were always 60% or less of those isolated from intact livers.

However, when the quantitative metabolic data are expressed per liver, certain differences are further accentuated. For example, estimations of the capacity of normal and regenerating livers to metabolize DMBA at various times after oil or DMBA injection are shown in Fig. 5. These values were calculated from the average activities of the various post-mitochondrial supernate fractions (displayed in Fig. 2) and the average yield of post-mitochondrial supernate protein per liver from each experimental group. When expressed in this way it can be seen that oil-injected intact livers had about 3 times more activity than oil-injected regenerating livers, when assayed 24–72 h after injection. Similarly, livers from DMBA-injected intact animals had a capacity to metabolize DMBA 3–5 times that of livers from similarly treated partially hepatectomized animals.

Calculations based on data from microsomes revealed more pronounced trends. Basal metabolic capacities of regenerating livers were 10–20% of those of intact livers, during the 24–72 h after oil injection. Likewise, livers from partially hepatectomized animals injected with DMBA had a capacity to metabolize DMBA about 25% that of livers isolated from DMBA-injected intact animals at early times after injection (data not shown).

The differences shown in Fig. 5 are clearly related to both the smaller size of regenerating livers during the first few days following partial hepatectomy and to the lower activities of regenerating liver cell fractions; the results indicate a greatly compromised capacity to metabolize

FIG. 5.—Estimated capacity of normal and regenerating livers to metabolize DMBA, based on post-mitochondrial supernate incubation data. Calculated from the average specific activities of the various post-mitochondrial supernate fractions shown in Fig. 2 and the average yield of post-mitochondrial supernate protein per liver from each experimental group.

DMBA during the early phases of liver regeneration.

Whether or not DMBA must be metabolically activated to become carcinogenic is uncertain at present. Although polycyclic hydrocarbons like benzo(a)-pyrene are metabolized by microsomal enzymes to highly reactive epoxides, which are more potent carcinogens than the parent compounds (Jerina and Daly, 1974; Heidelberger, 1975), evidence exists that DMBA is carcinogenic in the absence of oxidative metabolism (Marquardt and Heidelberger, 1972; Marquardt *et al.*, 1974). Also, a recent study showed that the K-region epoxide of DMBA failed to elicit sarcomas after injection into rats, whereas DMBA was highly tumorigenic (Flesher, Harvey and Sydnor, 1976). Thus, the decreased capacity of regenerating liver to metabolize DMBA may be an important factor in the susceptibility of this tissue to DMBA carcinogenesis.

The results described here may partly account for the persistence of unmetabolized DMBA in regenerating rat liver as previously reported (Tomsak and Cook, 1975) but other factors may be of importance. For example, fatty infiltration is a prominent morphological and biochemical feature of early liver regeneration, and is most conspicuous during the first 2 days following subtotal hepatectomy (Bucher and Malt, 1971). The composition of this material is mainly neutral lipid, and its source appears to be body fat (Bartsch and Gerber, 1966; Glende and Morgan, 1968). In addition, ultrastructural studies have demonstrated that at least some lipid enters regenerating hepatocytes by the process of pinocytosis (Trotter, 1965). It is also known that DMBA and other polycyclic hydrocarbons distribute to tissue fat in large quantities when administered to normal rats (Daniel, Pratt and Prichard, 1967). Thus, it is conceivable that the high lipid content of regenerating liver may serve as a depot for DMBA, and that augmented transport of DMBA from body fat to the regenerating liver may also be a factor in the increased persistence of DMBA during hepatic regeneration. Radioautographic studies designed to test some of these possibilities are presently under way in our laboratory.

We thank Mitchell Smith for reviewing the manuscript, and Dr John R. Carter for his continuing encouragement and support. Jean Pirina and Bonnie Lou Berry provided expert secretarial assistance.

This work was supported in part by American Cancer Society Institutional Grant No. IN57-L, and U.S.P.H.S. Pathobiology Training Grant No. 5 TO1 GMO1784–08.

### REFERENCES

BARKER, E. A., ARCASOY, M. & SMUCKLER, E. A. (1969) A Comparison of the Effects of Partial Surgical and Partial Chemical ($CCl_4$) Hepatectomy on Microsomal Cytochrome $b_5$ and $P_{450}$ and Oxidative N-Demethylation. *Agents and Actions*, **1**, 27.

BARTSCH, G. G. & GERBER, G. B. (1966) Incorporation of Acetate-1-$^{14}$C into Lipids by the Perfused Liver of Normal, X-irradiated, or Partially Hepatectomized Rats. *J. Lipid Res.*, **7**, 204.

BOOTH, J., KEYSELL, G. R. & SIMS, P. (1973) Formation of Glutathione Conjugates as Metabolites of 7,12-Dimethylbenz(a)anthracene by Rat-liver Homogenates. *Biochem. Pharmacol.*, **22**, 1781.

BOYLAND, E. & SIMS, P. (1967) The Effect of Pretreatment with Adrenal-protecting Compounds on the Metabolism of 7,12-Dimethylbenz(a)anthracene and Related Compounds by Rat-liver Homogenates. *Biochem. J.*, **104**, 394.

BUCHER, N. L. R. & MALT, R. A. (1971) *Regeneration of Liver and Kidney*. Boston: Little Brown.

CHERNOZEMSKI, I. N. & WARWICK, G. P. (1970) Liver Regeneration and Induction of Hepatomas in $B6AF_1$ Mice by Urethan. *Cancer Res.*, **30**, 2685.

CHIESARA, E., CONTI, F. & MELDOLESI, J. (1970) Influence of Partial Hepatectomy on the Induction of Liver Microsomal Drug-metabolizing Enzymes Produced by Phenobarbital: A Biochemical and Ultrastructural Study. *Lab. Invest.*, **22**, 329.

CRADDOCK, V. M. (1973) Induction of Liver Tumors in Rats by a Single Treatment with Nitroso Compounds Given after Partial Hepatectomy. *Nature, Lond.*, **245**, 386.

DANIEL, P. M., PRATT, O. E. & PRICHARD, M. M. L. (1967) Metabolism of Labelled Carcinogenic Hydrocarbons in Rats. *Nature, Lond.*, **215**, 1142.

FLESHER, J. W., HARVEY, R. G. & SYDNOR, K. L. (1976) Oncogenicity of K-Region Epoxides of Benzo(a)pyrene and 7,12-Dimethylbenz(a)anthracene. *Int. J. Cancer*, **18**, 351.

FOUTS, J. R., DIXON, R. L. & SHULTICE, R. W. (1961) The Metabolism of Drugs by Regenerating Liver. *Biochem. Pharmacol.*, **7**, 265.

GENTIL, A., LASNE, C. & CHOUROULINKOV, I. (1974) Metabolism of 7,12-Dimethylbenz(a)anthracene by Hamster Liver Homogenates. *Xenobiotica*, **4**, 537.

GLENDE, E. A. & MORGAN, W. S. (1968) Alteration in Liver Lipid and Lipid Fatty Acid Composition after Partial Hepatectomy in the Rat. *Exp. mol. Path.*, **8**, 190.

GRAM, T. E., GUARINO, A. M., GREENE, F. E., GIGON, P. L. & GILLETTE, J. R. (1968) Effect of Partial Hepatectomy on the Responsiveness of Microsomal Enzymes and Cytochrome P-450 to Phenobarbital or 3-Methylcholanthrene. *Biochem. Pharmacol.*, **17**, 1769.

HEIDELBERGER, C. (1975) Chemical Carcinogenesis. *Ann. Rev. Biochem.*, **44**, 79.

HENDERSON, P. T. & KERSTEN, K. J. (1970) Metabolism of Drugs During Rat Liver Regeneration. *Biochem. Pharmacol.*, **19**, 2343.

HIGGINS, G. M. & ANDERSON, R. M. (1931) Experimental Pathology of the Liver I. Restoration of Liver of White Rat Following Partial Surgical Removal. *Arch. Path.*, **12**, 186.

HILTON, J. & SARTORELLI, A. C. (1970) Induction by Phenobarbital of Microsomal Mixed Oxidase Enzymes in Regenerating Rat Liver. *J. biol. Chem.*, **245**, 4187.

JELLINCK, P. H. & GOUDY, B. (1967) Effect of Pretreatment with Polycyclic Hydrocarbons on the Metabolism of Dimethylbenzanthracene-12-$^{14}$C by Rat Liver and Other Tissues. *Biochem. Pharmacol.*, **16**, 131.

JELLINCK, P. H., SMITH, G. & FLETCHER, R. (1970) Nature of the Water-soluble Metabolites of 7,12-Dimethylbenz(a)anthracene Formed by Liver Microsomes of Normal and Methylcholanthrene Treated Rats. *Cancer Res.*, **30**, 1715.

JERINA, D. M. & DALY, J. W. (1974) Arene Oxides: A New Aspect of Drug Metabolism. *Science, N.Y.*, **185**, 573.

LEVIN, W. & CONNEY, A. H. (1967) Stimulatory Effect of Polycyclic Hydrocarbons and Aromatic Azo Derivatives on the Metabolism of 7,12-Dimethylbenz(a)anthracene. *Cancer Res.*, **27**, 1931.

MARQUARDT, H., STERNBERG, S. S. & PHILIPS, F. S. (1970) 7,12-Dimethylbenz(a)anthracene and Hepatic Neoplasia in Regenerating Rat Liver. *Chem.-Biol. Interact.*, **2**, 401.

MARQUARDT, H. & HEIDELBERGER, C. (1972) Influence of "Feeder Cells" and Inducers and Inhibitors of Microsomal Mixed Function Oxidases on Hydrocarbon Induced Malignant Transformation of Cells Derived from C3H Mouse Prostate. *Cancer Res.*, **23**, 721.

MARQUARDT, H., SODERGREN, J. E., SIMS, P. & GROVER, P. L. (1974) Malignant Transformation *In vitro* of Mouse Fibroblasts by 7,12-Dimethylbenz(a)anthracene and 7-Hydroxymethyl-12-methylbenz(a)anthracene and by their K-region Derivatives. *Int. J. Cancer*, **13**, 304.

NEBERT, D. W. & GEILEN, J. E. (1972) Genetic Regulation of Aryl Hydrocarbon Hydroxylase Induction in the Mouse. *Fed. Proc.*, **31**, 1315.

POUND, A. W. (1968) Carcinogenesis and Cell Proliferation. *N.Z. med. J.*, **67**, 88.

POUND, A. W. & LAWSON, T. A. (1974) Effects of Partial Hepatectomy on Carcinogenicity, Metabolism, and Binding to DNA of Ethyl Carbamate. *J. natn. Cancer Inst.*, **53**, 423.

POUND, A. W. & LAWSON, T. A. (1975) Partial Hepatectomy and Toxicity of Dimethylnitrosamine and Carbon Tetrachloride, in Relation to the Carcinogenic Action of Dimethylnitrosamine. *Br. J. Cancer*, **32**, 596.

SOEDIGDO, S., ANGUS, W. W. & FLESHER, J. W. (1975) High Pressure Liquid Chromatography of Polycyclic Aromatic Hydrocarbons and Some of Their Derivatives. *Anal. Biochem.*, **67**, 664.

SPENCER, T. & FISCHER, P. W. F. (1971) The Induction of Microsomal Hydroxylases in Regenerating Rat Liver. *Chem.-Biol. Interact.*, **4**, 41.

STOMING, T. A. & BRESNICK, E. (1974) Hepatic Epoxide Hydrase in Neonatal and Partially Hepatectomized Rats. *Cancer Res.*, **34**, 2810.

TOMSAK, R. L. & COOK, R. T. (1975) The Persistence of Unmetabolized $^{3}$H-7,12-Dimethylbenz(a)anthracene in Regenerating Rat Liver. *Br. J. Cancer*, **32**, 440.

TROTTER, N. L. (1965) Electron-Opaque, Lipid-Containing Bodies in Mouse Liver at Early Intervals after Partial Hepatectomy and Sham Operation. *J. Cell Biol.*, **25**, 41.

UESUGI, T., BOGNACKI, J. & LEVINE, W. G. (1976) Biliary Excretion of Drugs in the Rat During Liver Regeneration. *Biochem. Pharmacol.*, **25**, 1187.

VON DER DECKEN, A. & HULTIN, T. (1960) The Enzymatic Composition of Rat Liver Microsomes During Liver Regeneration. *Expl Cell. Res.*, **19**, 591.

WARWICK, G. P. (1971) Effect of the Cell Cycle on Carcinogenesis. *Fed. Proc.*, **30**, 1760.

WEISBURGER, J. H. & WILLIAMS, G. M. (1975) Metabolism of Chemical Carcinogens. In *Cancer: A Comprehensive Treatise Vol. 1.* Ed. F. F. Becker. N.Y.: Plenum Press.

YANG, S. K. & DOWER, W. V. (1975) Metabolic Pathways of 7,12-Dimethylbenz(a)anthracene in Hepatic Microsomes. *Proc. natn. Acad. Sci. U.S.A.*, **72**, 2601.